# RT-PCR cytokine study in patients with allergic rhinitis

**DOI:** 10.1016/S1808-8694(15)30827-2

**Published:** 2015-10-18

**Authors:** Tarcimara Moreira da Silva, Roberto Eustáquio Santos Guimarães, Evaldo Nascimento, Helena Maria Gonçalves Becker, Ricardo Nascimento Araújo, Flávio Barbosa Nunes

**Affiliations:** 1 MSc in Medicine, Otorhinolaryngologist, Professor - FEAD Centro de Gestão Empreendedora.; 2 PhD in Medicine, Associate Professor of Medicine - USP de Ribeirão Preto and Adjunct Professor of Otorhinolaryngology - Department of Otolaryngology, Speech and Hearing Therapy and Ophthalmology of the Medical School of the Federal University of Minas Gerais - FM-UFMG.; 3 PhD in Medicine, Adjunct Professor - Department of parasitology - ICB - UFMG.; 4 PhD in Medicine, Adjunct Professor - Department of Otolaryngology, Speech and Hearing Therapy and Ophthalmology of the Medical School of the Federal University of Minas Gerais - FM-UFMG.; 5 Veterinarian - Parasitology.; 6 PhD student - Graduate Program in General Surgery - Medical School - UFMG, Otorhinolaryngology.

**Keywords:** allergen, cytokines/immunology, hypersensitivity, rhinitis/allergic/perennial, rhinitis/allergic/seasonal, skin/tests

## Abstract

Allergic rhinitis is an inflammatory reaction of the nasal mucosa, in consequence of an IgE mediated hypersensitive reaction to inhaling allergens, involving different mediators and cytokine cells. **Aim:** The purpose of this study was to evaluate the transcriptions for IL-4, IL-5, IL-8 and IFN-gama, particularly important in the nasal allergy process, especially IL-4 and IL-5. For this study we decided to evaluate atopic patients who were free from allergic crises, with the purpose of knowing the cytokine expressions during this period. **Materials and Methods:** Another prospective and transversal study was carried out, selecting 30 patients, 13 of these patients were pauci-symptomatic and 17 were non atopic. The groups were selected by means of a medical interview, an otolaryngologic clinical exam and allergy skin tests - Prick Test. The cytokines were investigated in fragments of the nasal mucosa, using RT-PCR - chosen because it has good reproducibility and specificity. **Results:** IL-5, IL-8, IFN-gama cytokine values were kept homogeneous in relation to the control group. Only IL-4 presented significant statistic differences. **Conclusion:** Asymptomatic patients with allergic rhinitis presented with normalization of cytokine expression in the nasal mucosa, with exception of IL-4.

## INTRODUCTION

IL-4, IL-5, IL-8 and IFN-γ cytokines, especially IL-4 and IL-5, mediate and regulate immune and inflammatory reactions in allergic rhinitis. Considering this information and the line of research developed in the Otorhinolaryngological institution where the present investigation was carried out, we decided to investigate these substances between two groups: one with paucisymptomatic allergic rhinitis and a non-allergic control group.

Reverse transcriptase-polymerase chain reaction was the method of laboratorial analysis chosen because it has good reproducibility and specificity^1^, ^2^.

The goal of the present investigation was to analyze, quantify and compare the expressions of the afore-mentioned cytokines in paucisymptomatic patients with allergic rhinitis, comparing them with a non-atopic control group in order to study the relevance these substances have in those individuals out of the allergic spell period.

## MATERIALS AND METHODS

This is a prospective, cross-sectional study, carried out in a sample of patients with and without allergic rhinitis. The patients chosen were those with previous surgical indication for septoplasty, adenoidectomy and/or tonsillectomy associated with partial turbinectomy. The fragments were collected from February of 2004 to April of 2005.

This research project was approved by the Ethics in Medical Research Committee (COEP) on October 14, 2004, under protocol # ETIC 044/04. All the patients and their companions were duly explained about the study, its goals and methods. Those who agreed signed the informed consent form.

These patients responded to an interview, they underwent clinical otorhinolaryngological exam and skin allergy test, they were then broken down into two groups: one group of patients with allergic rhinitis, made up of 13 patients and another group made up of 17 non-atopic patients. The factors impacting sampling were: the high cost of the material utilized, the lack of trained professionals to carry out the exam (RT-PCR) and the long time it took to import the primers and the time set to conclude the study.

Age ranged between three and 47 years, and they were children and adults, regardless of race, gender, religion or social class. Among these patients, 16 underwent septoplasty, 12 underwent adenoidectomy and two suffered adenotonsillectomy. All these patients suffered turbinectomy, thus making up a total of 30 partial lower turbinates removed, which were then sent for cytokine analysis.

We took off the study all those patients under anti-histamine and steroid agents for at least thirty days before the turbinectomy to collect the nasal mucosa fragment, or that could not terminate the use of medication that impacted the skin test response, patients with severe skin eczema or dermatitis and patients below three years of age or elderly (above 60 years of age) because of a higher occurrence of false negatives due to a reduction in test reactivity in old age.

The patients were submitted to the prick test on the medial face of their forearms in adults and on the back in small children.

As antigen we have the following: D. farinae, D. pteronyssinus, house dust, Blatella sp, grass, fungi, feathers, cats and dogs. For positive control we used histamine and for the negative one we used saline solution^3^. This material was purchased from the International Pharmaceutica Immunology - Brazil Ltda Laboratories. (IPI) São Paulo.

Nasal mucosa fragments were placed in 1.5ml “Eppendorf” tubes, sterile, identified with the patient’s name and collection data. Afterwards, these tubes were placed in a Styrofoam box and taken to the Hospital’s Immunology Laboratory. There, the fragments were frozen at - 80°C.

RNA extraction was carried out through using Trizol® (Invitrogen) reagent according with the manufacture’s instructions. The tissues were macerated, and the resulting solution was transferred to 1.5ml tubes. In each sample we added 0.2 ml of chloroform, and after vigorous mixing, the solution was incubated in ice for 15 minutes, followed by centrifugation at 12,000 x g per 15 minutes at 4°C. The upper layer resulting from the centrifugation was then transferred to a new 1.5ml tube and the RNA was precipitated with isopropanol (Sigma). The RNA was then resuspended in 40 ml of MiliQ water (Millipore - Brazil) and treated with 2.5 U of DNAse: RNAse free (Promega) for 20 minutes at 37ºC. The RNA was re-extracted using 200 ml of Trizol®(Invitrogen) reagent and resuspended in 30 ml of MiliQ water. The RNA concentration was quantified in a spectrophotometer at 260nm wavelength and its integrity was assessed by denaturizing electrophoresis in 0.8% agarose gel.

The cDNA was synthesized from the 1.25 mg of total RNA using random hexamer primers (Promega) and the reverse transcriptase system - SuperScript II (Invitrogen) according to manufacture’s instructions. PCR was carried out in a final volume of 20ml, in the presence of 1ml of cDNA, 0.25mM of dNTP, 0.2 mM of each primer and 1 U of Platinum TaqDNA polymerase (Invitrogen).

PCR products were analyzed by electrophoresis in an 8% polyacrylamide gel dyed by silver. The gels were photographed and the results were analyzed by densitometry using AlphaDigiDoc 1201TM (AlphaInotech).

Since it was not possible to clone the genes and obtain the absolute quantity necessary for RT-PCR in real time, we used the semi-quantitative RT-PCR and the β-Actin (“housekeeping”) was considered the reference gene. The standard coefficient was then acquired from dividing the β-Actin value from each patient by the average of the 30 patients. This dosage was called relative because we measured the control expression in relation to the standard test^4^, ^5^, ^6^, ^7^.

In the statistical analysis, among the measures of interest, we used parametric and non-parametric tests, with statistical significance level fixed at 5% and a confidence interval at a minimum of 80% and a maximum of 95%.

Continuous variables such as age, IL-4, IL-5, IL-8, IFN-γ and β-Actin were analyzed as such, and central trend measures were calculated (mean and median) as well as scatter measures (standard deviation and amplitude)^8^, ^9^.

Variables gender, age and IL-4, IL-5, IL-8, IFN-γ cytokines were compared for both groups of patients: allergic and non-allergic and, through the statistical tests employed (Bartlett’s chi-squared, ANOVA, t-test, Kruskal Wallis’s test and Pearson’s correlation), there was no statistical difference, except for IL-4, which had a p<0.05.

## RESULTS

The age of the patients in the study varied between three and 47 years, and Table 1 shows the mean, median, standard deviation and range.

**Table 1:** 

	Group	Mean	Median	Standard Deviation	Range
Age	Allergic	13,4	11	7,4	3-26
	Non-allergic	19,2	19	13	3-47

p value				0,21	

Age distribution in the two groups was asymmetrical because the patients involved in this study were those with indication for surgeries that could provide fragments of the nasal mucosa.

There was no significant statistical difference among the ages of the two groups, p=0.21.

Table 2 shows the Pearson’s correlation coefficient used to compare β-Actin, IL-4, IL-5, IL-8 and IFN-γ with the age variable. We did not observe any statistically significant correlation among the variables presented.

**Table 2:** Pearson’s correlation coefficient between age and response variables.

Independent variable	r	r2	p value
b-actin	-0,264	0,070	0,158
IL-4	-0,178	0,032	0,348
IL-5	-0,341	0,116	0,065
IL-8	-0,236	0,056	0,210
IFN-g	-0,182	0,033	0,337

r : Pearson’s correlation coefficient.

r² : Determination coefficient.

In the group of allergic patients, 23% of them were females and 77% were males. In the non-allergic group, 53% were females and 47% were males. We did not see any racial or gender preference associated with this disease^10^, ^11^, ^12^.

All the patients complained of nasal obstruction because they presented diseases that also blocked their upper airways, such as adenoid hypertrophy and/or palatine tonsil hypertrophy, obstructive nasal septum deviation and nasal concha hypertrophy.

Patients from the atopic group were paucisymptomatic in relation to their allergy when they were assessed in the preoperative; however, a history of mucosal hyperactivity was present, which is a clinical landmark of allergic rhinitis. In the allergy test, there was a predominance of sensitivity towards D. farinae(100% of the patients) followed by D.pteronyssinuse house dust (84%). Grass was sensitizing-antigen in 46% of the patients and sensitivity towards Blatella sp, dogs and fungi were similar (23%). No patient was sensitive to feathers.

Comparisons among variables IL-4, IL-5, IL-8 and IFN-γ were adjusted by β-Actin value among allergic and non-allergic patients (Table 3). IL-4 was the only cytokine to have a statistically significant increase (p=0.03). IL-5 had a strong trend towards increasing; however without statistically significant difference (p=0.06). Values for IL-8 and IFN-γ were similar in both groups: IL-8 had a p=0.17 and IFN-γ had a p=0.72.

**Table 3:** Comparison between interleukins and interferon corrected by actin among allergic and non-allergic patients.

Allergic					Non-allergic				
Variable	Mean	SD	Median	Range	Mean	SD	Median	Range	p value
b-actin	80209	10221	75909	66332-96794	80552	11467	77443	64111-99882	0,98
IL-4	37894	38998	24133	2838-126110	13396	15577	4624	119-51296	0,03
IL-5	39966	29899	30124	3013-92463	18714	17334	11382	2875-71402	0,06
IL-8	44515	23349	40622	17418-95491	37491	33518	27079	6887-129924	0,17
IFN-g	123144	72907	99817	29679-184417	102575	42430	95574	29679-184417	0,72
Age	13,2	7,8	11,0	3,0-26,0	21,2	15,5	19,0	3,0-58,0	0,21

## DISCUSSION

We did not see a statistically significant difference for the age variable (p=0.21) between the groups. Age was compared with each cytokine and no statistically significant correlation was found. As far as gender is concerned, in the group of allergic patients, 23% were females and 77% were males. Although this study’s sample was small, it confirms reports from the II Brazilian Consensus on Rhinitis (2006)^13^, and although allergic rhinitis is a common disease, we did not find real epidemiological data because most studies about its rate of occurrence and the diversity of associations investigated are in regards of data obtained only one time, and usually from small samples.

All the patients complained of nasal obstruction, and they also had diseases that blocked their upper airways, such as: adenoid and/or tonsil hypertrophy, nasal septum deviation and nasal turbinate hypertrophy, known causes of upper airway obstruction^14^.

Patients with allergic rhinitis were paucisymptomatic in the preoperative and their medical history led us to believe they had mild to moderate persistent rhinitis according to the Allergic Rhinitis and Its Impact on Asthma (ARIA) initiative and that of the World Health Organization^13^.

According to Mion and Mello Jr. (2006)^3^, sensitizing antigens of allergic rhinitis that most occur in our settings com from house mites and other components of house dust. Therefore, in Brazil, the clinical symptoms of patients with allergic rhinitis are frequent throughout the entire year, but they increase during fall/winter seasons, because during this time climatic conditions favor house mite proliferation, although this ectoparasite is present in people’s homes throughout the year. The results from the present study are in agreement with the data present in the literature^3^, ^13^.

The values for IL-4, IL-5, IL-8 and IFN-γ were analyzed and standardized through the data obtained and adjusted by the β-actin component, similar to the pattern used by Mamoni and M.H. Botta (2005)^2^. The semiquantitative reverse transcriptase polymerase chain reaction (RT-PCR) was a potentially useful method, bringing subsidies for the study of citokines in allergic patients, having seen that this disease is a good model to study these substances, according to Naggar et al.(1998)^15^.

IL-8 and IFN-γ results were similar in both groups, except for IL-4 and there was a strong trend towards IL-5 increase.

In 1997, a study carried out by Lee et al.(1997)^16^ involving 20 patients (10 patients with perennial allergic rhinitis who were not submitted to nasal provocation and 10 control patients showed that IL-4 and IL-5 were universally expressed in atopic patients. Thus, supporting our study, where IL-4 was the cytokine that had a significant increase (p=0.03), indicating that there are differences between the paucisymptomatic allergic and non-allergic groups. This cytokine, because it has a greater participation in allergenic sensitization, was found in high levels; however, it was not associated with clinical manifestations, contributing to maintain nasal inflammation.

IL-5 levels have a tendency to increase, but there was no statistically significant difference (p=0.06). Ohashi at al. (1998)^17^ compared the levels of IgE, IL-5 and IFN-γ in three groups: non-atopic, asymptomatic atopic and symptomatic atopic. They did not see a significant difference in IL-5 levels in the non-atopic and asymptomatic atopic patients. Similarly, the paucisymptomatic allergic patients of this study had IL-5 levels similar to those of non-atopic patients. On the other hand, these authors observed a significant relation among the increased IL-5 levels, nasal eosinophilic infiltration and the clinical manifestations of allergic rhinitis. Asakura et al. (1998)^18^ blocked IL-5 effects with monoclonal antibody (anti-IL-5), consequently inhibiting eosinophilic, histamine hypersensitivity and nasal symptoms. These values can be persistently increased in allergic individuals, and this requires further studies^16^.

For authors such as Benson et al. (2000)^19^ IFN-γ has an important function in allergic disease. It is a cytokine produced by Th1 cells, inhibiting Th2 response. According to his studies, an impaired release of IFN-γ, hypothetically the basic trace of atopy, could lead to the development of atopy, allowing the Th2 response to induce an increase in the levels of intranasal IgE in atopic patients. Bottcher et al.(2002)^20^, studying a group of children, observed that those who developed allergic rhinitis were the ones who had a reduction in IFN-γ levels. In our study we noticed that there was no statistically significant difference in the IFN-γ levels between the two groups investigated. According to Naggar et al.,(1998)^15^, IgE production is closely related with a balance between IL-4 (Th2 response) and IFN-γ (Th1 response). In their study with sensitized rats, there was a predominance of Th2 response and an increase in IL-4 levels, with reduction in IFN-γ (Th1) levels, in agreement with our results.

IL-8 is one of the many citokines^21^ with the function of reducing leukocyte migration^22^ and the production of leukotrienes. It is an important cytokine in the late phase of the allergic reaction, especially in the release of histamin^23^. According to Ohkubo et al.(1997)^24^, the levels, the sources and the release mechanisms of this cytokine can vary, but it is important in allergic rhinitis manifestations.

In our study, with a sample of 30 patients, it was not possible to state that there was a difference in comparing IL-8 and IFN-γ among allergic and non-allergic patients, because the comparison power between the mean and standard deviation values was always below 80%. If the study had been made with 133 allergic patients and 133 non-allergic patients for IFN -γ values, and 266 allergic and 266 non-allergic patients for IL-8, we would reach a power of 80%, then, we could be sure whether or not the groups were similar. Even then, without the statistical difference, this study is in agreement with the literature, there is no significant expression of IFN-γ, since it is reduced in atopic patients and IL-8, more frequently associated with the clinical manifestations of the late phase of the allergic disease, which did not characterize the group being investigated.

## CONCLUSION

In comparing cytokine profiles between non-allergic patients and paucisymptomatic patients with perennial allergic rhinitis during natural exposure to allergens, without provocation, we observed an increase in IL-4 expression, a trend towards IL-5 expression increase, Th2-type citokines, no statistically significant difference in the expression of IFN-γ and Th-1-type cytokine, thus suggesting that the Th2-type response is crucial for the physiopathology of allergic rhinitis in vivo.

## References

[1] Barbuto JAM (2001). Imunidade celular, in Imunologia.

[2] Mamoni RL, Blotta MH (2005). Kinetics of cytokines and chemokines gene expression distinguishes Paracoccidioides brasiliensis infection from disease. Cytokine.

[3] Mion O, Mello Jr JF (2006). Up-to-date em Rinite. Diagnóstico das Rinites.

[4] Mygind N, Bretlau P (1974). Scanning electron microscopic studies of the human nasal mucosa in normal persons and in patients with perennial rhinitis. II. Secretion. Acta Allergol Denmark.

[5] Mygind N, Winther B (1979). Light and scanning electron-microscopy of the nasal mucosa. Acta Otorhinolaryngol Belg.

[6] Tengowski MW, Feng D, Sutovsky M, Sutovsky P (2007). Differential expression of genes encoding constitutive and inducible 20S proteasomal core subunits in the testis and epididymis of the ophylline or 1,3-dinitrobenzene-exposed rats. Biol Reprod.

[7] Bryan D, Walker KB, Ferguson M, Thorpe R (2005). Cytokine gene expression in a murine wound healing model. Cytokine.

[8] Fonseca JS, Martins GA (1996). Curso de estatística.

[9] Goulart EMA (2000). Metodologia e Informática na Pesquisa Médica.

[10] Araújo CAF, Barros LF, Miranda Jr MAB, Fontes RS (2003). Rinopatia alérgica: Conduta terapêutica adaptada para países em desenvolvimento, in Revista da Sociedade de Otorrinolaringologia do Estado do Rio de Janeiro.

[11] Van Cauwenberge P, Bachert C, Passalacqua G, Bousquet J, Canonica GW, Durham SR (2000). Consensus statement on the treatment of allergic rhinitis. European Academy of Allergology and Clinical Immunology. Allergy.

[12] Van Hoecke H, Vastesaeger N, Dewulf L, De Bacquer D, van Cauwenberge P (2006). Is the allergic rhinitis and its impact on asthma classification useful in daily primary care practice?. J Allergy Clin Immunol.

[13] Solé D, Mello Jr JF, Weckx LLM, Rosário Filho NA, Cruz AA, Campos CAH (2006). II Consenso Brasileiro sobre Rinites 2006. In Rev Bras Alerg Imunopatol.

[14] Cruz OL, Costa SS (1994). Otorrinolaringologia: princí-pios e prática.

[15] Naggar MM, K Ukai K, Takeuchi & Sakakura Y (1998). Expression of Interferon-gama, Interleukin-4and Interleukin-5 mRNA in the Nasal Mucosa Membrane of Rats with Allergic Rhinitis. Scand J Immunol.

[16] Lee CH, Rhee CS, Oh SH, Min YG, Lee MS (1997). Increase in expression of IL-4and IL-5 mRNA in the nasal mucosa of patients with perennial allergic rhinitis during natural allergen exposure. Ann Otol Rhinol Laryngol.

[17] Ohashi Y, Nakai Y, Tanaka A, Kakinoki Y, Masamoto T, Kato A (1998). Allergen-induced synthesis ofinterleukin5, but not of IgE, is a key mechanism linked to symptomatic episodes of seasonal allergic rhinitis in sensitized individuals. Scand J Immunol.

[18] Asakura K, Saito H, Watanabe M, Ogasawara H, Matsui T, Kataura A (1998). Effectsof anti-IL-5 monoclonal antibody on the murine model of nasal allergy. Int Arch Allergy Immunol.

[19] Benson M, Strannegard IL, Wennergren G, Strannegard O (2000). Low levels of interferon-gamma in nasal fluid accompany raised levels of T-helper 2cytokines in children with on going allergic rhinitis. Pediatr Allergy Immunol.

[20] Bottcher MF, Jenmalm MC, Bjorksten B (2002). Immune responses to birch in young children during their first 7 years of life. Clin Exp Allergy.

[21] Leonard EJ, Skeel A, Yoshimura T, Noer K, Kutvirt S, Van Epps D (1990). Leucocyte specificity and binding of human neutrophil attractant/ activation protein-1. J Immunol.

[22] Dahinden CA, Kurimoto Y, De Weck AL, Lindley I, Dewald B, Baggiolini M (1989). Theneutrophil-activating peptide NAF/NAP-1induces histamine and leukotriene release by interleukin 3-primedbasophils. J Exp Med.

[23] Bischoff SC, Brunner T, De Weck AL, Dahinden CA (1990). Interleukin modifies histamine release and leukotriene generation by human basophils in response to diverse agonist. J Exp Med.

[24] Ohkubo K, Ikeda M, Pawankar R, Gotoh M, Yagi T, Okuda M (1998). Mechanisms of IL-6, IL-8, and GM-CSF release in nasal secretions of allergic patients after nasal challenge. Rhinology.

